# Effects of omalizumab on chronic urticaria not responding to recommended therapy

**DOI:** 10.1186/1710-1492-8-S1-A17

**Published:** 2012-11-02

**Authors:** Jacques Hébert, Rose-Marie Caron-Guay

**Affiliations:** 1 Centre de recherche appliquée en allergie de Québec, Québec City, Canada; 2 Allergie et Immunologie, CHUQ/Université Laval, Québec City, Canada

## Background

Treatment of chronic urticaria consists of antihistamines as the first-line treatment. For more severe symptoms, combinations can be necessary as well as dose augmentations. The recent guidelines suggest the possibility of using omalizumab in resistant cases. We treated 2 patients with cold-urticaria (CO), 1 with cholinergic urticaria(CH) and 11 with chronic spontaneous urticaria (CSU) with omalizumab, who had not benefited from the recommended first-line, second-line and third-line treatments.

## Methods

Patients were required to document their CU symptoms once daily with urticaria activity scores for 7 days (7UAS). Briefly, the symptoms were monitored in terms of numbers of wheals [none (=0 points), <10 (=1 point), 10–50 (=2 points), or >50 per day (=3 points)], and the intensity of their pruritus [none (=0 points), mild (=1 point), moderate (=2 points), severe (=3 points)], for a total of 42 points. To evaluate the efficacy of the omalizumab treatment, 7UAS obtained at baseline was compared to that at the third and sixth month of the therapy. Omalizumab was given at 150 μg/month irrespective of IgE levels and increased to 300 mg if needed (no response). The concomitant medication was slowly reduced according to clinical response.

## Results

**Figure 1:**
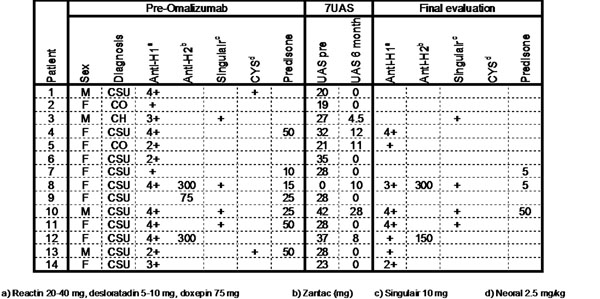


The 7UAS improved significantly in all severe urticaria patients with omalizumab as early as one month (not shown) after initiation of therapy and was sustained for the 6 month observation (Figure 1). 7UAS in patient #8 was >30 previously but =0 at entry because treated with oral prednisone for >1year. The response was not satisfactory for patient # 10 and omalizumab increased to 300 mg after 6 months with a better clinical response (not shown). Along with the clinical improvement, the concomitant medications could also be reduced significantly in all patients except #10, particularly prednisone.

## Conclusion

Our results show that omalizumab improves significantly recommended treatment-resistant urticaria patients (13/14) in terms of clinical symptomatology (7UAS) and drug reduction in a real life setting. None of the patients reported any adverse effect.

